# Treatment patterns and outcomes in patients with non‐small cell lung cancer receiving biosimilar filgrastim for prophylaxis of chemotherapy‐induced/febrile neutropaenia: Results from the MONITOR‐GCSF study

**DOI:** 10.1111/ecc.13034

**Published:** 2019-04-10

**Authors:** Matti Aapro, Andriy Krendyukov, Nadja Höbel, Pere Gascon

**Affiliations:** ^1^ Cancer Center Clinique de Genolier Genolier Switzerland; ^2^ Hexal AG Holzkirchen Germany; ^3^ Division of Medical Oncology, Department of Hematology‐Oncology, Hospital Clínic de Barcelona University of Barcelona Barcelona Spain

**Keywords:** biosimilar, filgrastim, granulocyte colony‐stimulating factor, non‐small cell lung cancer, real world

## Abstract

**Objective:**

Real‐world evidence data on the use of granulocyte colony‐stimulating factor (G‐CSF) in patients with non‐small cell lung cancer (NSCLC) are limited. MONITOR‐GCSF is a pan‐European, multicentre, prospective, non‐interventional study designed to describe patient characteristics, treatment patterns and clinical outcomes in patients receiving biosimilar filgrastim in the prophylaxis of chemotherapy‐induced neutropaenia (CIN) and febrile neutropaenia (FN).

**Methods:**

In this subanalysis, patient characteristics, treatment patterns, and outcomes are described for 345 patients with stage 3 or 4 NSCLC, receiving up to six chemotherapy cycles. Patients were treated with biosimilar filgrastim as per their treating physician's best judgement.

**Results:**

CIN (any grade) occurred in 13.6% of patients in Cycle 1 and in 36.5% of patients in all cycles. FN occurred in 1.4% of patients in Cycle 1 and in 5.2% of patients in all cycles. Grade 3–4 FN occurred in 1.2% of patients in Cycle 1 and in 3.8% of patients in all cycles.

**Conclusion:**

Results show that in real‐life practice in patients with NSCLC, biosimilar filgrastim has similar effectiveness and safety to the known effectiveness and safety profile of reference filgrastim, supporting the use of biosimilar filgrastim for the real‐world treatment of neutropaenia in patients with NSCLC.

## 1 INTRODUCTION

Lung cancer is one of the leading causes of cancer‐related mortality worldwide, accounting for around 1.3 million deaths annually (Vansteenkiste et al., 2013). Non‐small cell lung cancer (NSCLC) is the most common histological type of lung cancer, accounting for 85%–90% of all cases (Montesinos et al., 2011; Novello et al., 2016). Approximately 381,500 patients are diagnosed with NSCLC in Europe each year (Montesinos et al., 2011), over two‐thirds of these with advanced disease (Davies et al., 2017).

Platinum‐based chemotherapy is the standard of care for NSCLC. Platinum doublets chemotherapy is recommended for adjuvant chemotherapy in stage II (A/B) and stage III (A/B) NSCLC (Postmus et al., 2018). Cisplatin‐based regimens (e.g., cisplatin/etoposide or cisplatin/vinorelbine) in conjunction with radiotherapy are also recommended in the treatment of locally advanced stage III NSCLC (Vansteenkiste et al., 2013). For advanced NSCLC, the current standard of care for first‐line treatment of stage IV epidermal growth factor receptor (EGFR) and anaplastic lymphoma kinase (ALK)‐negative disease comprises platinum doublets, with carboplatin‐based doublet chemotherapy recommended for elderly patients (Planchard et al., 2018). For tumours with an activating EGFR mutation, first‐line treatment with an EGFR tyrosine kinase inhibitor (gefitinib, erlotinib, afatinib, dacomitinib, and orosimertinib) is recommended, with ALK inhibitors (crizotinib, alectinib, ceritinib, and brigatinib) preferred for patients with ALK‐rearranged NSCLC (NCCN, 2018; Planchard et al., 2018). Survival in patients with NSCLC remains poor, with 5‐year survival rates estimated at 10%–20% (Davies et al., 2017). Platinum‐induced myelosuppression is a complication associated with chemotherapy in NSCLC patients (Cao et al., 2016). Some regimens (i.e., carboplatin/docetaxel, cisplatin/etoposide and cisplatin/vinorelbine/cetuximab) are associated with a high risk of chemotherapy‐induced neutropaenia (CIN)/febrile neutropaenia (FN) (Aapro et al., 2011; Crawford, Caserta, & Roila, 2010). This can result in dose reductions and/or delays to chemotherapy, which can impact on treatment success (Aapro et al., 2011). Prophylactic prevention of CIN/FN is therefore warranted to ensure cytotoxic chemotherapy is delivered on time and at efficacious doses (Rivera, Haim Erder, & Fridman, 2003). International guidelines recommend primary prophylaxis with granulocyte colony‐stimulating factors (G‐CSFs), such as filgrastim or pegfilgrastim, for patients with a 20% or greater risk of CIN/FN (i.e., those receiving a high‐risk chemotherapy regimen, or those with risk factors that may increase the overall CIN/FN risk) (Aapro et al., 2011; Crawford, Caserta, & Roila 2010; Smith et al., 2006,2015). Filgrastims have demonstrated efficacy in decreasing the incidence, severity and duration of CIN/FN episodes, and reducing the risk of dose reduction, discontinuation or delays to chemotherapy (Aapro et al., 2016).

The use of G‐CSF prophylaxis varies widely in real‐life clinical practice, both in the timing of therapy and the type of patients who use it (Aapro et al., 2011). Furthermore, G‐CSF prophylaxis is often not used in line with guideline recommendations (Krzemieniecki et al., 2014). Further data on the efficacy and safety of G‐CSF prophylaxis and its use in a real‐world setting are needed.

MONITOR‐GCSF is an international, multicentre, prospective, open‐label, non‐interventional study of cancer patients treated with myelosuppressive chemotherapy regimens whose treating physicians prescribed CIN/FN prophylaxis with Sandoz biosimilar filgrastim (Zarzio^®^/Zarxio^®^/EP2006; Hexal AG/Sandoz International GmbH). Treatment patterns and outcomes associated with CIN/FN prophylaxis with biosimilar filgrastim in 1,447 patients with solid or haematologic malignancies have previously been reported (Gascón et al., 2016). In this paper, we describe patient characteristics, treatment patterns, and outcomes for the cohort of patients with NSCLC from MONITOR‐GCSF who received primary or secondary prophylaxis with biosimilar filgrastim as part of routine clinical practice.

## 2 METHODS

### 2.1 Design

The background and methodology of MONITOR‐GCSF have previously been described (Gascón, Aapro, Ludwig, Rosencher, Boccadoro et al., 2011; Gascón, Aapro, Ludwig, Rosencher, Turner et al., 2011). In brief, MONITOR‐GCSF was a European‐wide, prospective, non‐interventional, multi‐level, pharmaco‐epidemiological study of chemotherapy‐treated cancer patients who started treatment with Sandoz biosimilar filgrastim for the prophylaxis of CIN/FN as per their prescribing physician's best clinical judgment. Male or female adults (aged ≥18 years) diagnosed with stage III or IV breast cancer, bladder cancer, NSCLC, or diffuse large B‐cell lymphoma, or metastatic prostate cancer were eligible for inclusion if they were scheduled to receive their first of at least four cycles of chemotherapy and received treatment with biosimilar filgrastim as indicated. The study was approved by the ethical review committees of participating centres in accordance with national laws and regulations. Patients provided written informed consent.

### 2.2 Data collection

Patients were observed for up to six cycles of chemotherapy. All available data were recorded. Descriptive data on demographics, clinical status, medical history, concomitant comorbid conditions and current status of disease, and prior and concomitant medications were collected at enrolment. Data on chemotherapy regimen, including any changes, were collected at every visit. Outcomes of interest included the incidence of CIN/FN, antibiotic prophylaxis, biosimilar filgrastim prophylaxis, and adverse events (AEs). Data were summarised overall and according to age ≥65 years and ≥70 years at baseline. Here we present results for patients with NSCLC only.

## 3 RESULTS

### 3.1 Patients

A total of 345 patients with NSCLC were included in MONITOR‐GCSF. Of these, 101 patients (29.3%) had stage III disease, and 241 (69.9%) had stage IV disease. Most patients (*n* = 240, 69.6%) were men. Mean body weight was 72.3 kg (range: 40.9–123 kg). Mean age was 62.9 years (range: 40–86 years), and overall, 142 patients (41.2%) were aged ≥65 years and 81 patients (23.5%) were aged ≥70 years.

Monotherapy regimens used by patients included docetaxel in 14 patients (4.1%) and topotecan in 11 patients (3.2%) (Table 1). The most commonly prescribed combination chemotherapy regimen was cisplatin/etoposide in 74 patients (21.4%). Other combination regimens included carboplatin/etoposide in 46 patients (13.3%), carboplatin/paclitaxel in 25 patients (7.2%), cisplatin/gemcitabine in 18 patients (5.2%), cisplatin/vinorelbine in 18 patients (5.2%), cisplatin/paclitaxel in nine patients (2.6%), and carboplatin/docetaxel in eight patients (2.3%).

**Table 1:** Chemotherapy regimens prescribed

Chemotherapy regimen	All patients (*n* = 223)	≥65 years of age (*n* = 78)	≥70 years of age (*n* = 42)
Monotherapy regimens			
Docetaxel	14 (4.1%) [2.40%; 6.70%]	6 (4.2%) [1.95%; 8.91%]	
Topotecan	11 (3.2%) [1.79%; 5.62%]		
Combination regimens			
Cisplatin/etoposide	74 (21.4%) [17.44%; 26.08%]	22 (15.5%) [10.46%; 22.43%]	11 (13.6%) [7.76%; 22.70%]
Carboplatin/etoposide	46 (13.3%) [10.15%; 17.33%]	17 (12%) [7.61%; 18.34%]	14 (17.3%) [10.58%; 26.95%]
Carboplatin/paclitaxel	25 (7.2%) [4.96%; 10.48%]	11 (7.7%) [4.38%; 13.34%]	6 (7.4%) [3.44%; 15.23%]
Cisplatin/gemcitabine	18 (5.2%) [3.33%; 8.10%]	7 (4.9) [2.41%; 9.83%]	6 (7.4%) [3.44%; 15.23%]
Cisplatin/vinorelbine	18 (5.2%) [3.33%; 8.10%]	10 (7%) [3.87%; 12.48%]	5 (6.2%) [2.67%; 13.65%]
Cisplatin/paclitaxel	9 (2.6%) [1.38%; 4.88%]		
Carboplatin/docetaxel	8 (2.3%) [1.18%; 4.51%]	5 (3.5%) [1.51%; 7.98%]	

Cisplatin/etoposide was the most commonly prescribed chemotherapy regimen in patients aged ≥65 years (22 patients, 15.5%), followed by carboplatin/etoposide (17 patients, 12%) and carboplatin/paclitaxel (11 patients, 7.7%). Carboplatin/etoposide was the most commonly prescribed chemotherapy regimen in patients aged ≥70 years (14 patients, 17.3%), followed by cisplatin/etoposide (11 patients, 13.6%) and cisplatin/gemcitabine or carboplatin/paclitaxel (each six patients, 7.4%).

### 3.2 Prophylaxis

Data on biosimilar filgrastim prophylaxis were available for 341 patients with NSCLC, including 142 aged ≥65 years and 81 aged ≥70 years (Table 2).

**Table 2:** Mean dose in patients with NSCLC included in the MONITOR‐GCSF study

Patient group	Mean dose (µg)	Median dose (min, max)	Discontinued prophylaxis, *n* (%)
All patients (*n* = 341)	6,962.0	6,000 (480; 40,320)	65 (19.1) [15.25%; 23.57%]
≥65 years of age (*n* = 142)	6,069.3	4,800 (480; 31,680)	27 (19.0) [13.41%; 26.25%]
≥70 years of age (*n* = 81)	5,820.0	4,500 (480; 31,680)	14 (17.3) [10.58%; 26.95%]

### 3.3 Clinical outcomes

A total of 126 (36.5%; 95% CI [32.00%; 42.19%]) patients experienced one or more CIN (any grade) episode and 18 (5.2%; 95% CI [3.36%; 8.19%]) patients had FN (any grade) throughout the duration of the study. In Cycle 1, CIN (any grade) occurred in 47 (13.6%; 95% CI [10.53%; 17.85%]) patients and FN occurred in 5 (1.4%; 95% CI [0.63%; 3.39%]) patients. Grade 3 or 4 FN occurred in four patients (1.2%; 95% CI [0.46%; 2.98%]) in Cycle 1 and in 13 patients (3.8%; 95% CI [2.24%; 6.41%]) in all cycles.

Changes to the chemotherapy regimen are detailed in Table 3.

**Table 3:** Change to chemotherapy regimen in patients with NSCLC included in the MONITOR‐GCSF study

Change to chemotherapy regimen, *n* (%)	All patients (*n* = 345)	≥65 years of age (*n* = 142)	≥70 years of age (*n* = 81)
Any change	276 (80.0) [75.46%; 83.88%]	119 (83.8) [76.87%; 88.96%]	68 (84.0) [74.45%; 90.37%]
Received <6 cycles	220 (63.8) [58.57%; 68.66%]	97 (68.3) [60.26%; 75.39%]	57 (70.4) [59.69%; 79.21%]
Dose reduced	47 (13.6) [10.40%; 17.65%]	21 (14.8) [9.88%; 21.55%]	11 (13.6) [7.76%; 22.70%]
Cycle delayed	77 (22.3) [18.24%; 27.00%]	34 (23.9) [17.67%; 31.59%]	18 (22.2) [14.54%; 32.42%]
Cycle cancelled	23 (6.7) [4.48%; 6.66%]	8 (5.6) [2.88%; 10.72%]	4 (4.9) [1.94%; 12.02%]

### 3.4 Safety

Adverse events reported in patients with NSCLC included arthralgia, bone pain, cough, gastroenteritis, and myalgia (each in one patient, 0.3%; 95% CI [0.05%; 1.64%]; Table 4).

**Table 4:** AEs reported in patients with NSCLC included in the MONITOR‐GCSF study

AE	Patients, *n* (%)
Arthralgia	1 (0.3) [0.05%; 1.62%]
Bone pain	1 (0.3) [0.05%; 1.62%]
Cough	1 (0.3) [0.05%; 1.62%]
Gastroenteritis	1 (0.3) [0.05%; 1.62%]
Myalgia	1 (0.3) [0.05%; 1.62%]

## 4 DISCUSSION

Evidence suggests that G‐CSF is frequently not used according to international recommendations in daily clinical practice, highlighting a need for further data from observational studies to help guide optimal use of G‐CSF. This may be particularly important in older patients who are at high risk of CIN/FN. This subanalysis described patient characteristics, treatment patterns, and clinical outcomes for patients with NSCLC who received primary or secondary prophylaxis with biosimilar filgrastim as part of routine clinical practice in the MONITOR‐GCSF observational study.

Of the patients with NSCLC included in MONITOR‐GCSF, patients had a mean age of 62.9 years, with 41% of patients aged ≥65 years and 24% of patients aged ≥70 years. The most commonly prescribed treatment regimen in the overall NSCLC population was cisplatin/etoposide, with carboplatin/etoposide the most common regimen in patients aged ≥65 and ≥70 years. It should be noted that etoposide is not a recommended component of a platinum‐based doublet regimen in the latest international published guidelines for NSCLC treatment. Due to the observational nature of MONITOR‐GCSF, it is important to highlight that real‐world practice does not always reflect guideline recommendations. Nonetheless, it should be considered that use of G‐CSF was appropriate in this group since the risk of FN in patients with NSCLC receiving cisplatin/etoposide is 54% (Font et al., 1999). Data are not available in the literature regarding the risk of FN in patients with NSCLC receiving carboplatin/etoposide.

A large proportion of patients (≥80%) experienced changes to their chemotherapy regimens—in addition to reasons such as lack of efficacy, tolerability concerns, and disease progression, chemoresistance may have been responsible for this finding since this is a widely reported limitation of cisplatin (Brabec, Kasparkova, Kostrhunova, & Farrell,^2016^ ). This highlights a need for improved understanding of the molecular mechanisms of cisplatin resistance and of identifying markers of resistance (Fennell et al., 2016).

Clinical outcomes for patients with NSCLC were generally consistent with those reported for the overall population of 1,447 patients, and for different patient populations included in MONITOR‐GCSF such as DLBCL and breast cancer, supporting clinical extrapolation (Aapro, Krendyukov, Krivtsova, & Gascón, 2018; Gascón et al., 2016; Gascón, Krendyukov, Höbel, & Aapro, 2018). Regarding CIN, 36.5% of NSCLC patients experienced one or more episode of any grade throughout the study, compared with 34.8% in the overall study population. In MONITOR‐GCSF, the percentage of patients experiencing FN (any grade) in patients with NSCLC (5.2%) was comparable to the overall population (5.9%). In Cycle 1, FN was reported in 1.4% patients with NSCLC, a similar level to the overall population in the HEXAFIL study (1.8%), an observational study assessing use of biosimilar filgrastim in routine clinical practice in Germany (Tesch et al., 2015). IMPACT Solid was a large prospective observational study designed to describe FN incidence and adherence to G‐CSF guidelines in patients with solid tumours with a FN risk of ≥20%. In Cycle 1, FN was reported in 4% of the 224 patients with NSCLC included in the study (Krzemieniecki et al., 2014). A retrospective analysis, including patients with breast cancer, colorectal cancer, and NSCLC receiving G‐CSF, aimed to evaluate the effect of G‐CSF on FN (McCune et al., 2012). However, only 18 FN events occurred in the 1,042 patients with NSCLC so the authors concluded that the effect of G‐CSF on FN could not be evaluated (McCune et al., 2012). These very limited data emphasise the need for long‐term observational studies in patients with NSCLC receiving G‐CSF, particularly those >65 years of age.

Adverse events were reported in five patients with NSCLC and included arthralgia, bone pain, cough, gastroenteritis, and myalgia (each in one patient, 0.3%). This is a lower level than in the overall population where 53.7% of patients experienced AEs (Gascón et al., 2016). The reasons for this are unclear and are worthy of further investigation. AEs for the NSCLC patients were not reported in the IMPACT Solid study. It should be noted that collection of AE data in non‐interventional studies is a widely recognised challenge since patients appear less likely to report well‐known side effects in this setting (Gascón et al., 2013).

The findings from this subanalysis of patients with NSCLC demonstrate that the efficacy and safety of biosimilar filgrastim in real‐life practice are similar to the known efficacy and safety profile of reference filgrastim. This supports the use of filgrastim biosimilar in patients with NSCLC in a real‐world setting and extends the efficacy and safety from its clinical development programme. The large percentage of patients aged ≥65 years included in the study adds to the body of evidence on how to best treat older patients with NSCLC receiving myelosuppressive chemotherapy.

## CONFLICT OF INTEREST

AK: Hexal AG (employment). MA: Amgen (honoraria, speakers bureau, expert testimony), Helsinn Healthcare (advisory role, speakers bureau, research funding), Hospira (advisory role, speakers bureau, research funding), Teva (advisory role, speakers bureau), Merck KGaA (advisory role), Merck (advisory role), Sandoz (advisory role, speakers bureau, research funding), Pierre Fabre Medicament (advisory role, speakers bureau, research funding), Vifor Pharma (advisory role, speakers bureau), Tesaro (advisory role, speakers bureau), Novartis (speakers bureau, research funding), Roche (speakers bureau), Johnson & Johnson (speakers bureau). NH: at the time of manuscript development Hexal AG (employment). PG: Sandoz (speakers bureau).
